# TNF-α gene polymorphisms and expression

**DOI:** 10.1186/s40064-016-3197-y

**Published:** 2016-09-07

**Authors:** Radwa R. El-Tahan, Ahmed M. Ghoneim, Noha El-Mashad

**Affiliations:** 1 Zoology Department, Faculty of Science, Damietta University, P.O. 34517, New Damietta, Damietta Egypt; 2 Clinical Pathology Department, Faculty of Medicine, Mansoura University, Mansoura, Egypt

**Keywords:** TNF-α, Polymorphisms, Expression, Autoimmune diseases

## Abstract

Tumor necrosis factor alpha (TNF-α) is a proinflammatory cytokine with an important role in the pathogenesis of several diseases. Its encoding gene is located in the short arm of chromosome 6 in the major histocompatibility complex class III region. Most of the TNF-α gene polymorphisms are located in its promoter region and they are thought to affect the susceptibility and/or severity of different human diseases. This review summarizes the data related to the association between TNF-α gene and its receptor polymorphisms, and the development of autoimmune diseases. Among these polymorphisms the −308G/A TNF-α promotor polymorphism has been associated several times with the the development of autoimmune diseases, however some discrepant results have been recorded. The other TNF-α gene polymorphisms had little or no association with autoimmune diseases. Current results about the molecules controlling TNF-α expression are also presented. The discrepancy between different records could be related partly to either the differences in the ethnic origin or number of the studied individuals, or the abundance and activation of other molecules that interact with the TNF-α promotor region or other elements.

## Background

Tumor necrosis factor (TNF), first termed by O’Malley et al. (1962), was initially reported to induce programmed cell death or apoptosis. Currently, this molecule is thought to be involved in the regulation of many important cellular processes such as proliferation, differentiation, growth, and the immune response (Hayashi et al. 2013). TNF-α is produced by various types of cells including macrophages, monocytes, neutrophils, T cells, and NK-cells. The gene encoding TNF-α is located in the class III region of the major histocompatibility complex on chromosome 6 between the HLA-B and HLA-DR genes (Zhang et al. 2013). TNF binds to two types of outer membrane bound receptors on the target cells, TNFR1 and TNFR2, and triggers the cell survival and proinflammatory NF-κB and MAP kinases activations (Locksley et al. 2001). The molecule activates phagocytes to engulf and clear infectious agents and cellular debris (Elahi et al. 2009). It also increases the expression of adhesion molecules on the vascular endothelium to allow immune cells, in particular neutrophils and macrophages, to translocate to the sites of tissue damage and infection (Barbara et al. 1996).

The roles that TNF-α play seem to be contradictory and this was related to the genetic polymorphisms in the genes regulating its production and effect (Elahi et al. 2009), and the polymorphisms in TNF locus itself. It has been reported that the genetic alterations in the TNF-α locus are involved in high TNF-α production (Tsukamoto et al. 1998).

Several TNF-α polymorphisms have been identified inside the TNF-α promoter at the positions, relative to the transcription start site, −1031 (T/C), −863 (C/A), −857 (C/A), −851 (C/T), −419 (G/C), −376 (G/A), −308 (G/A), −238 (G/A), −162 (G/A), and −49 (G/A) (Elahi et al. 2009).

In this article, we review the association between the genetic polymorphisms in TNF-α and the development of autoimmune diseases, and the relation between these polymorphisms and TNF-α expression.

## Polymorphisms in TNF-α receptors

TNF-α interacts with the TNF receptors TNF-RI and TNF-RII (Bayley et al. 2003). TNF-α receptors (TNF-Rs) are active both in membrane-bound and soluble forms, and the soluble receptors act as physiological attenuators of TNF activity (Aderka 1996). With respect to their chromosomal location, TNF-RI gene is located at 12p13 and the TNF-RII gene is located at 1p36.2 (Bayley et al. 2003).

With respect to the relation between TNF-Rs and autoimmune diseases, no association was reported between TNF-RI +36 and rheumatoid arthritis (RA) in Dutch and UK Caucasian population as approved by Bayley et al. (2003) and Barton et al. (2001) respectively. No association was found between the TNF-RII +1690 polymorphism and susceptibility to or severity of RA in the Dutch population (Bayley et al. 2003).

However, TNF-RII 196M/R SNP was found to be associated with susceptibility to familial RA (Barton et al. 2001; Dieude et al. 2002), but not associated with sporadic RA severity in Caucasian patients (Van der Helm-van Mil et al. 2004). Glossop et al. (2003) and Constantin et al. (2004) have reported conflicting results about considering TNF-RII 196 M/R SNP as a genetic factor in RA severity. The 676TT genotype of TNF-α RII was related to a better response to anti-TNF-*α* drugs when compared to 676TG (Ongaro et al. 2008).

## Association between TNF-α genetic polymorphisms and autoimmune diseases

### Systemic lupus erthymatosus (SLE)

In SLE patients, an increased level of TNF-α was reported and strongly correlated with the parameters of disease activity (Studnicka-Benke et al. 1996). A significant genetic association between TNF-α promoter −308A/G polymorphism and SLE susceptibility in Asian populations, and in European-derived populations was detected in Zou et al. (2011) and Lee et al. (2006) meta-analysis respectively.

The −308A allele of TNF-α was associated with the production of TNF-α and susceptibility to SLE (Rood et al. 2000; Sullivan et al. 1997). This allele contributed to susceptibility to SLE in South African patients (Wang et al. 1999).

Piotrowski et al. (2015) indicated the presence of significant contribution of TNF-α −308 A allele to arthritis and renal SLE manifestation, and that the TNF-α −308 G/A polymorphism may be a HLA-DRB1*03:01 haplotype-dependent genetic risk factor for SLE in a cohort of Polish population.

The +489A allele of TNF-α was also thought to have a genetic contribution to the susceptibility to SLE in the Chinese population (Lin et al. 2009).

### Rheumatoid arthritis (RA)

TNF-α is thought to play a central role in inflammation and it has been directly implicated in the pathogenesis of RA (Feldmann et al. 1990). High concentrations of TNF-α were detected in serum and synovial fluid of RA patients and TNF-α blood concentration correlated with RA disease activity (Nemec et al. 2008).

Studies on the association between −238G/A and −308G/A TNF polymorphisms and Juvenile Idiopathic Arthritis (JIA) showed conflicting results. For example, the −238 G/A polymorphism did not have an effect on the patients’ outcome in either Turkish or Czech patients while the −308 G/A polymorphism was significantly associated with a poor outcome in the Turkish group but not in the Czech patients (Ozen et al. 2002) or with systemic JIA in Japanese population (Date et al. 1999). However, TNF-α −308A allele was significantly associated with JIA (JimÊnez-Morales et al. 2009), with systemic JIA (Modesto et al. 2005). Date et al. (1999) demonstrated that the −863A, −1013C, and −857T alleles were significantly higher in systemic JIA Japanese patients.

TNF-*α* was also considered as a major candidate gene in psoriatic arthritis (PsA) (Kane and FitzGerald 2004) as TNF-*α* was present in high levels in serum, synovial fluid, and synovial membrane in patients with PsA (Danning et al. 2000; Partsch et al. 1997). However, studies on the association between TNF-*α* polymorphisms and PsA showed conflicting results. A strong association between −308G/A and −238G/A promoter polymorphisms and susceptibility to PsA was demonstrated in some studies (Mössner et al. 2005; Rahman et al. 2006). In contrast, Murdaca et al. (2014) did not find significant association between −238 and −308G/A SNPs and PsA in Italian patients but they found a significant association between +489A allele and both PsA susceptibility and severity. Rahman et al. (2006) could not detect any significant association between −1031T/C, −863C/A and −857C/T SNPs and PsA.

Also, studies on the relation between TNF-α polymorphisms and RA showed conflicting results. For example, susceptibility to RA was associated with the −308A allele in some studies (JimÊnez-Morales et al. 2009; Lee et al. 2007), with the G allele in others (Mosaad et al. 2011), but neither with A allele nor G allele in others (Ates et al. 2008; Gambhir et al. 2010; Khanna et al. 2006; Nemec et al. 2008; Rezaieyazdi et al. 2007).

Heterozygous genotype GA of −308A/G SNP was found to be associated with more severe course of RA disease (Cvetkovic et al. 2002), with increased number of erosions, and with the progression of radiographic damage in patients with early seropositive RA (Khanna et al. 2006). RA severity was associated with the presence of −308A allele (Mosaad et al. 2011; Rodríguez-Carreón et al. 2005) and with −308G allele (Nemec et al. 2008). The −308G allele showed a trend toward worse radiological outcome by 5 years in patients with inflammatory arthritis as indicated by the presence/absence of erosions (Barton et al. 2004). In Han Chinese population, susceptibility of RA increased in patients with TNF-α −308G allele, especially in the females, and the patients containing both HLA-DRB1*04 and TNF-α–308 GG genotype showed a significant increase in risk for RA regardless of their sex (Li et al. 2015). On the other hand, in a cohort of Argentinean patients with RA, the −308A allele was neither associated with suscetibility to RA nor with the course and outcome of the disease (Aranda et al. 2014). In addition, there was no significant association between −308 G/A polymorphism and RA risk in a cohort of Bulgarian population (Manolova et al. 2014).

−238G/A polymorphism was associated with more severe course of RA (Fabris et al. 2002), however, Barton et al. (2004) showed that both −238G/A and −376G/A were not associated with RA severity. No association was reported between −238G/A and −376G/A polymorphisms and susceptibility to RA in Egyptian population (Mousa et al. 2014). Barton et al. (2004) failed to find any difference in either allele or genotype frequences of −1031, −863, −857, +489, +851 and +1304 SNPs between patients with inflammatory arthritis developing erosions and those remaining non erosive at 5 years.

Fonseca et al. (2007) demonstrated an association between −238, −308, −857, and −863 SNPs and systemic manifestations, functional status, radiological damage, work disability, and joint surgeries.

### Ankylosing spondylitis (AS)

TNF-α level in blood and its expression by peripheral T cells correlated well with AS activity (Bal et al. 2007; Rudwaleit et al. 2001). Studies on the relation between TNF-α SNPs and AS have shown controversial results. For example, TNF-α polymorphisms had no independent effect on AS susceptibility (Chung et al. 2011; Li et al. 2010) but their modulating effect on TNF-α expression were well relevant to the phenotypic diversity in AS (Lee and Song 2009; Poddubnyy et al. 2011). In contrast, Vargas-Alarcón et al. (2006) and Shiau et al. (2007) showed an association of −308G/A polymorphism with susceptibility to AS. Moreover, the A allele was thought to have a protective role against AS (Chung et al. 2011; Nossent et al. 2014), and was associated with a lower risk of developing AS, and with the age at disease onset, disease severity and response to anti-TNF treatment (Manolova et al. 2014).

In conclusion, the data from the studies on TNF-α genetic polymorphisms seem to vary from one study to another. This variation could be related to the differences in the ethnic orgin or the number of the indivisulas under study. Although these polymorphisms do affect the expression level of TNF-α, the activation and abundance of other molecules that interact directly or indirectly with the promotor sequence must affect the expression of TNF- α.

## Control of TNF-α gene expression

**Fig. 1:**
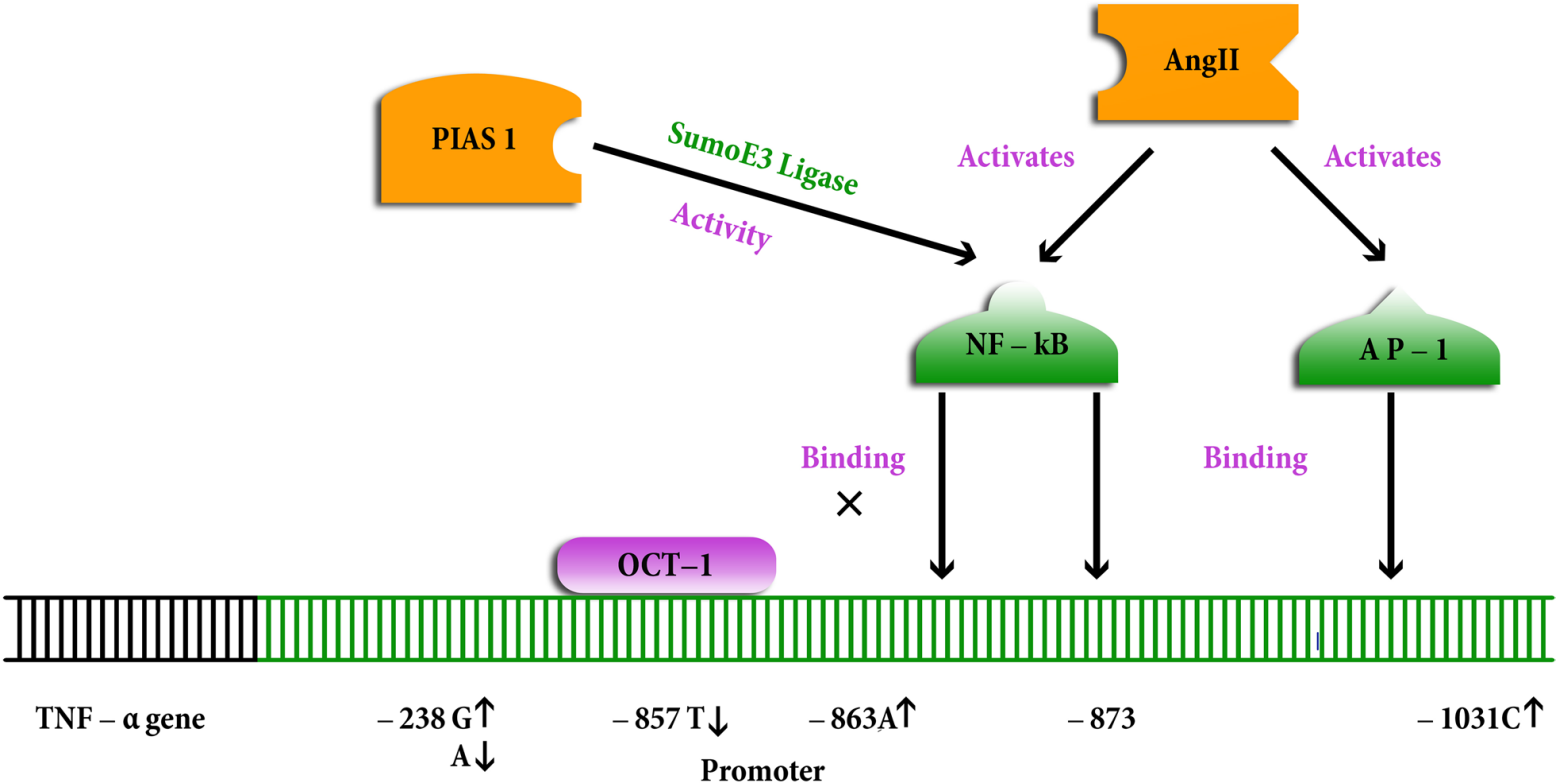
Schematic representation showing some of the molecules thought to be involved in the interaction with TNF-α promoter region. The transcriptional induction of TNF-α is thought to be controlled by some transcription factors, including the transcription factor OCT1, the nuclear factor κB (NF-κB), the signal transducer and transcription activator (PIAS1) and activator protein-1 (AP-1). The transcription factor OCT1 can strongly bind with the allele -857T (but not the -857C) and thus blocks the interaction of NF-κB to the nearby region -873 to -863 leading to inhibition of TNF-α transcription. PIAS1 possesses SUMO E3 ligase activity and can repress NF-κB by blocking the DNA-binding activity of NF-κB to TNF-α promoter. Ang II can activate the 2 transcription factors NF-kB and AP-1that are important in mediating TNF-α gene expression. Alleles associated with upregulation of TNF-α gene are designated with arrows with heads directed up while those alleles associated with downregulation are designated with arrows with heads directed down. Molecules involved in the posttranscriptional and posttranslational control of TNF-α are mentioned in the text and not shown here

Several studies tried to address the relation between TNF-α polymorphisms and its expression, and the mechanisms controlling its expressions in many cell types and diseases. High TNF-α expression level was associated with the −238G allele in multiple sclerosis patients (Huizinga et al. 1997) and with −863A and −1031C alleles in healthy Japanese and non-Japaneses individuals (Higuchi et al. 1998). While the −238A allele was reported to down regulate TNF-α expression (Kaluza et al. 2000). In appearently healthy individuals, van Heel et al. (2002) demonstrated that the −857T (but not the −857C) allele inhibits TNF-α transcription through its strong binding with the transcription factor OCT1, which blocks the interaction of nuclear factor-kappa-B (NF-κB) to the nearby region −873 to −863 (Fig. 1).

Mousa et al. (2014) detected a significant increase of TNF-α expression in RA patients compared to healthy individuals, but this increase in expression was not linked to a certain allele of the −238G/A and −376G/A SNPs. Besides, several polymorphisms in some TNF-linked genes were also thought to regulate the expression of TNF directly or indirectly (Abraham and Kroeger 1999).

According to Liu and Shuai (2008), TNF-α can be autoregulated by activating PIAS1 [a member of the protein inhibitor of activated STAT1 (signal transducer and activator of transcription 1) family] SUMO E3 ligase. It is though that once activated, PIAS1 is then recruited to the TNF-α gene promoter to repress transcription (Liu and Shuai 2008).

The neurohormonal molecule, angiotensin II (Ang II), was thought to play a role in up regulation of TNF-α gene expression as Ang II activates two transcriptional factors that are important in mediating TNF-α gene expression, nuclear factor (NF)-_k_B and activator protein-1 (AP-1) (Chua et al. 1998; Hernandez-Presa et al. 1997; Ruiz-Ortega et al. 1998) (Fig. 1). Angiotensin II also provokes TNF biosynthesis in various nonmyocyte cell types (Ferreri et al. 1998; Klahr and Morrissey 1998).

Oxidized low density lipoprotein (LDL) and LDL were demonstrated to decrease TNF mRNA expression in NK cells (Malorni et al. 1997). Mitogen-activated Protein Kinase (MAPK) has been found to play a central role in the induced TNF-α expression in monocytes, macrophages, mast cells and T cells (Hoffmeyer et al. 1999; Ishizuka et al. 1997; Swantek et al. 1997; Zhang et al. 1997; Zhu et al. 2000).

The 3′ UTR of TNF-α contains a sequence element that is though to affect posttranslational control of TNF through mRNA stability and translation efficiency (Elahi et al. 2009). TNF mRNA has two protein-binding regions (Hel et al. 1998, 1996) located in the AU-rich element (ARE) within 3′ UTR (Garnon et al. 2005). Both AREs were reported to interact with several proteins including TIAR and AUF1 (DeMaria and Brewer 1996; Gueydan et al. 1999) which control TNF mRNA post transcription. Pituitary adenylate cyclase-activating polypeptide is another protein that was found to inhibit TNF-α expression (Manecka et al. 2014).

cAMP has been reported to play an important role in regulating TNF-α expression, for example the elevation of cellular cAMP suppresses TNF-α production (Gobejishvili et al. 2006; Zidek 1999).

Interferon regulatory factor-5 (IRF5) has been also reported to play a key role in the induction of TNF-α (Barnes et al. 2002). Krausgruber et al. (2010) has reported that TNF secretion in human monocyte-derived dendritic cells is mediated by cooperative action of IRF5 and RelA at the 5′ upstream and 3′ downstream regions of the TNF gene.

In conclusion, the expression of TNF-α seems to be controlled by the activation of other cellular molecules including signal transducer molecules, nuclear factors and second messenger molecules. More studies to address the interaction between these molecules and TNF-α are required to finally map a TNF-α pathway. Profiling the expression of the key molecules inside such pathway can open an avenue to control TNF-α over or downregulation.

## Conclusion

TNF-α plays an important role in the pathogenesis of autoimmune diseases. Several studies show that TNF-α gene promoter polymorphisms affect the susceptibility and/or severity of autoimmune diseases. Polymorphisms in the TNF receptors does not seem to be associated with the development of autoimmune diseases. The −308 G/A TNF-α promotor polymorphism seems to be highly associated with the development of these diseases, however some discrepant results have been recorded. Other TNF-α gene polymorphisms had little or no association with autoimmune diseases. This discrepancy might be explained by the differences in the ethnic orgin or number of the studied individuals. TNF-α gene expression is controlled by the presence of some polymorphisms in its promoter region and by several types of signalling molecules or nuclear factors that interact with the TNF-α promotor region or other elements.

## Abbreviations

Ang II: angiotensin II
ARE: AU-rich elements
AS: ankylosing spondylitis
AUF1: AU-rich element RNA-binding *protein* 1
HLA-B: human leukocyte antigen-B
HLA-DR: human leukocyte antigen-DR
JIA: juvenile idiopathic arthritis
LDL: low density lipoprotein
MAP kinases: mitogen-activated protein kinases
NF-κB: nuclear factor kappa-light-chain-enhancer of activated B cells
NK-cells: natural killer cells
OCT1: organic cation transporter 1
PIAS 1: protein inhibitor of activated STAT-1
STAT-1: signal transducer and activator of transcription-1
PSA: psoriatic arthritis
RA: rheumatoid arthritis
SLE: systemic lupus erthymatosus
SNPs: single nucleotide polymorphisms
SUMO E3 ligase: small ubiquitin-like modifier E3 ligase
TNFR-I: tumor necrosis factor receptor-1
TNF-α: tumor necrosis factor alpha
TIAR: T-cell-restricted intracellular antigen 1-related protein
